# Tracking Thermo-Oxidation Reaction Products and Pathways of Modified Lignin Structures from Reactive Molecular Dynamics Simulations

**DOI:** 10.1021/acs.jpca.4c00964

**Published:** 2024-06-25

**Authors:** S. Ahmed, S. J. Eder, N. Dörr, A. Martini

**Affiliations:** † Department of Mechanical Engineering, University of California Merced, 5200 N. Lake Road, Merced, California 95343, United States; ‡ AC2T research GmbH, Viktor-Kaplan-Straße 2/C, 2700 Wiener Neustadt, Austria; § Institute of Engineering Design and Product Development, TU Wien, Lehárgasse 6 − Objekt 7, 1060 Vienna, Austria

## Abstract

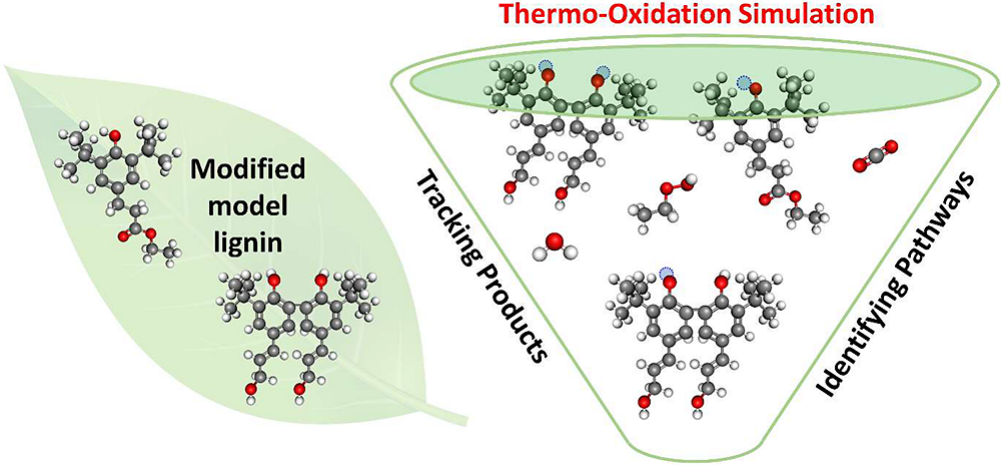

Thermo-oxidation of biomass is an important process that occurs through a variety of reaction pathways depending on the chemical nature of the molecules and reaction conditions. These processes can be modeled using reactive molecular dynamics to study chemical reactions and the evolution of converted molecules over time. The advantage of this approach is that many molecules can be modeled, but it is challenging to use the large amount of data obtained from such a simulation to determine reaction products and pathways. In this study, we developed a tracking approach to identify the reaction pathways of the dominant reaction products from reactive molecular dynamics simulations. We demonstrated the approach for thermo-oxidation reactions of modified model lignin compounds. For two modified lignin structures, we tracked the evolving chemical species to find the most common reaction products. Subsequently, we monitored specific bonds to determine the individual steps in the reaction process. This combined approach of reactive molecular dynamics and tracking enabled us to identify the most likely thermo-oxidation pathways. The methodology can be used to investigate the thermo-oxidative pathways of a wider range of chemical compounds.

## Introduction

Lignocellulose is a highly complex plant-based biomass that is a promising alternative resource for producing fuels and chemicals due to its abundant and renewable nature.^1^ The primary components of lignocellulosic biomass are cellulose, hemicellulose, and lignin. The process of converting lignocellulosic biomass into ethanol utilizes cellulose and hemicelluloses, leaving lignin as a waste.^2^ Significant quantities of lignin are also produced as a byproduct in the pulp and paper industry.^3^ However, lignin is currently underutilized because of its complex polymeric structure. It is built from phenolic precursors, i.e., lignols or monolignols, that randomly couple to form highly heterogeneous polymers. Polymerization is catalyzed by oxidative enzymes and occurs via the double bonds and hydroxy groups found in coniferyl alcohol, sinapyl alcohol, and paracoumaryl alcohol, which are the main types of lignols.^4^ However, lignin is inherently resistant to chemical and biological degradation,^5^ which makes it difficult to extract or modify without specialized processes and technologies.

The chemical structure of lignin suggests that it may be a good source of valuable chemicals after depolymerization and conversion.^6^ Depolymerization is a process that converts the polymers into their monomers. Conversion modifies the lignin structure by introducing various moieties into the structure, aiming to generate value-added useful chemicals. There are numerous methods for lignin depolymerization and conversion that can be broadly categorized as thermochemical, biological, and microwave-assisted^2,7^ as well as electrochemical.^8,9^ One of the thermochemical methods for depolymerization and conversion of lignin is thermo-oxidation.^2^

Thermo-oxidation is used to convert a wide range of biomass resources, including lignin, into high-yield products.^10^ Oxidation can prompt the cleavage of side chains, resulting in the production of phenolic aldehydes and acids, and it also has the capacity to cleave the aromatic rings in the lignin network, leading to the formation of aliphatic carboxylic acids.^11,12^ Researchers have studied the oxidation products of lignin to better understand its chemical transformations. For example, one study showed that the alkaline oxidation of softwood lignin produced vanillin and vanillic acid, whereas hardwood lignin yields syringaldehyde and syringic acid.^13^ The potential for acetic acid production through the wet oxidation of lignin has also been studied.^14^ To fully understand these chemical transformations, it is necessary to reveal the sequential series of steps leading to the formation of products from reactants, i.e., the reaction pathway. Mass spectrometry, especially high-resolution techniques, and subsequent data analysis have recently enabled progress in lignin analysis.^4^ However, it is not only difficult to reconstruct reaction pathways from the reaction products found, monitoring individual molecules requires significant effort. Therefore, identifying reaction pathways using only experimental methods is challenging.

Molecular dynamics (MD) simulations can complement experiments to investigate dynamic processes and reactions at the molecular level. In the case of lignin, the complex, heterogeneous nature of the biopolymer is challenging to simulate, so researchers often use model compounds. A model compound of lignin is a simplified chemical compound that represents a specific structural unit to focus on specific bonds or functional groups found in natural lignin. There are generally two types of lignin model compounds: monomers and dimers. These compounds have an aromatic structure and are characterized by functional groups such as hydroxyl, methoxyl, and alkyl.^15^ In previous studies, MD simulations were carried out to capture the movement, interactions, and conformational evolution of lignin with a nonreactive force field. Such nonreactive MD simulations of lignin structure in an aqueous solution revealed that, with decreasing temperature, lignin molecules undergo a transition from a state that is mobile and extended to one that is glassy and compact.^16^ In a similar study, a softwood and a hardwood lignin model were built from 61 monomer units each and then solvated with 30,500 water molecules to examine diffusion in various water models.^17^ Most recently, another study utilized nonreactive MD simulations to predict the solubility parameters of lignin in ionic liquids.^18^ However, nonreactive MD cannot provide bond dissociation, which is necessary for simulating the thermo-oxidation of lignin.

The reactive molecular dynamics approach can effectively capture the dynamic processes of bond breaking and formation within complex molecular systems using reactive force fields such as ReaxFF.^19^ Reactive MD simulations have shown great promise for elucidating chemical reactions. For instance, a previous study demonstrated the reaction pathways that yielded cresol as the predominant reaction product in the thermal decomposition of tricresyl phosphate on various ferrous surfaces using reactive MD.^20^ Another reactive MD-based study investigated ethanol oxidation in the presence of aluminum nanoparticles and illustrated the mechanisms of ethanol combustion with aluminum nanoparticle additives.^21^ However, there have been a few studies exploring the thermo-oxidation of lignin or lignin-based materials using this approach. One such study utilized reactive MD methods to investigate thermal decomposition in an oxygen environment for models representing the most prevalent linkages found in softwood, revealing the reaction pathways of dominant reaction products.^22^ The same author identified chemical transformations that convert flexible linkages in dilignol model compounds to rigid cyclic connections using reactive MD.^23^ However, the identified reaction pathways were determined visually, leading to the possibility that they may not represent the statistically dominant pathways.

In this study, we analyzed the thermo-oxidation of a modified lignin monomer and a dimer using reactive MD simulations. These compounds were obtained by introducing various moieties to create distinct lignin-based molecules by hydrogenation, esterification, and butylation. We developed a statistically robust method to track reaction products and identify pathways through a quantitative analysis of bond breaking and formation. The new tracking approach was demonstrated for thermo-oxidation of two model lignin-based compounds and enabled the identification of the most likely reaction pathways.

## Methods

**Figure 1:**
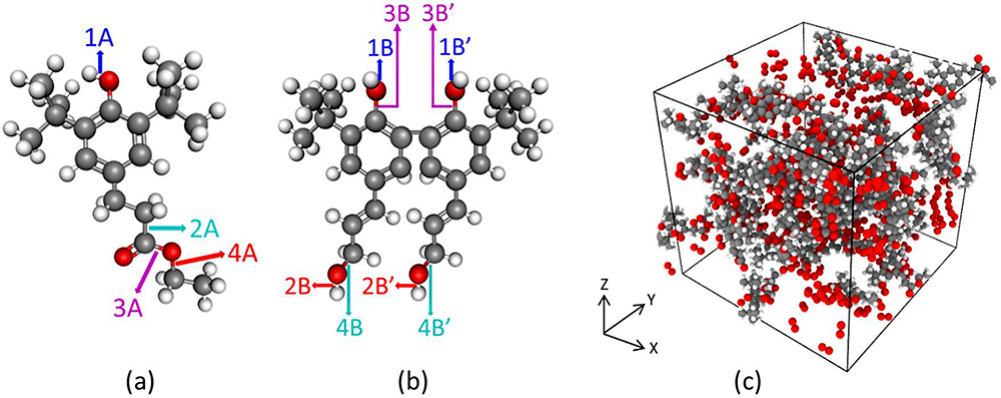
Chemical structures of molecules (a) A and (b) B with relevant bonds numbered. (c) Representative snapshot of the initial configuration of a simulation box with 50 molecules of type A and 300 O_2_ molecules. Gray, white, and red atoms correspond to carbon, hydrogen, and oxygen, respectively.

Reactive MD simulations were used to model thermo-oxidation of two molecules ethyl 3-(3,5-di-*tert*-butyl-4-hydroxyphenyl)propanoate and 3,3′-di-*tert*-butyl-5,5′-di-[(1E)-3-hydroxy-1-propen-1-yl](1,1′-biphenyl)-2,2′-diol, subsequently referred to as molecules A (C_19_H_30_O_3_) and B (C_26_H_34_O_4_), as shown in Figure 1a,b. These molecules were selected due to their potential synthesis from lignin monomers using processes such as hydrogenation, esterification, and butylation. Additionally, they have structural similarities with oxygen inhibitor additives used in lubricants, a subject we plan to explore in future research. BIOVIA Materials Studio^24^ was used to construct the initial structure of each molecule. These initial structures were then energy-minimized using the same software. Finally, 50 model molecules and 300 O_2_ were placed randomly in a simulation box. The large number of O_2_ molecules relative to the number of reactive sites on the model molecules was used to accelerate reactions such that they could be analyzed within the short time scale of the simulation. Figure 1c depicts the simulation box with initial dimensions of approximately 4 × 4 × 4 nm^3^ for molecule A and O_2_, with a total atom count of 3200. For the system with 50 molecule B and 300 O_2_, the simulation box contained a total of 3800 atoms. Periodic boundary conditions were applied in all three directions.

All simulations were performed using the Large Atomic/Molecular Massively Parallel Simulator (LAMMPS) software^25^ with the ReaxFF potential. ReaxFF is a reactive force field that models chemical reactions by allowing atoms to form and break bonds.^26^ It utilizes bond-order-based interactions, which include bond energies, torsion angles, and other valence terms, allowing it to dynamically adjust as molecular structures evolve during reactions. ReaxFF also includes nonbonded interactions such as van der Waals and Coulomb forces, applied to each pair of atoms ensuring that both short-range and long-range interatomic forces are considered, as well as a shielding function to moderate short-range interactions. ReaxFF force fields are parametrized by fitting parameters to experimental and quantum mechanical training sets.^27,28^ We used the ReaxFF force field parametrization that was originally developed for modeling the interactions between butane and O_2_ with a pyrite-covered Cr_2_O_3_ catalyst.^29^ The force field not only contains the necessary parameters for C, H, and O but also offers the possibility of future simulations that incorporate metal catalysts. There are numerous studies that used this force field for the thermal decomposition and oxidation of hydrocarbons.^30,31^ The dynamic simulations had a time step of 0.25 fs. Temperature regulation was accomplished through a Berendsen thermostat^32^ with a damping parameter of 25 fs. For pressure control, we adopted the Nosé–Hoover barostat^33,34^ with a damping parameter of 250 fs.

Every simulation started with an initial energy minimization, followed by dynamic equilibration. After energy minimization, equilibration was performed using the canonical ensemble (NVT with Berendsen thermostat: constant number of atoms *N*, volume *V*, and temperature *T*) at 300 K for 200 ps, followed by the isothermal–isobaric ensemble (NPT: constant number of atoms *N*, pressure *P*, and temperature *T*) at 300 K and 1 atm for 1 ns. Therefore, overall, the duration of the initial equilibration was 1.2 ns. The NVT stage allows the molecules to reorient into a stable energy configuration in a fixed, large-volume box, and then, the NPT simulation allows the volume of the box to change as the system approaches the density corresponding to ambient pressure and temperature. The duration of the equilibration steps was determined based on the time required to reach stable potential energy during NVT and stable density during NPT.

After the system equilibration, temperature ramp simulations were run to determine the temperature at which degradation begins, i.e., the first occurrence of dissociation of chemical bonds within a molecule. The temperature was increased from 300 to 3500 K over a period of 800 ps in the NVT ensemble, corresponding to a heating rate of 4 K/ps. Our preliminary study suggested that the degradation onset decreased with decreasing heating rate, but, once the heating rate was low enough, further reducing the rate did not significantly change the onset temperature. Therefore, we selected the heating rate of 4 K/ps because it was high enough to be achievable with our computational resources while also being low enough to ensure minimal dependence of the onset temperature on the heating rate. The heating process was repeated three times for each model system starting from the same equilibrated configuration but with different initial atom velocity distributions.

Finally, thermo-oxidation simulations were run at a constant temperature, starting with a configuration taken from each of the three temperature ramp simulations for both molecules A and B. The three independent simulations for each molecule provided more data from which reaction products and pathways could be analyzed. These simulations were carried out using the NVT ensemble and ran for 1 ns. The duration was determined in a series of preliminary simulations to be long enough to capture a sufficient number of reactions for robust analysis of products and pathways.

Throughout the simulations, atom trajectories and bond order data were output every 1000 timesteps, i.e., every 250 fs, resulting in a total of 4000 data sets per repeat simulation. The bond order data and connectivity information were used as input for custom Python scripts to detect molecules based on connectivity between atoms and record what and how many of each species were present. The logic behind this is described in more detail in the next section. Additionally, OVITO^35^ was utilized for visualizing atomic trajectories.

## Results

**Figure 2:**
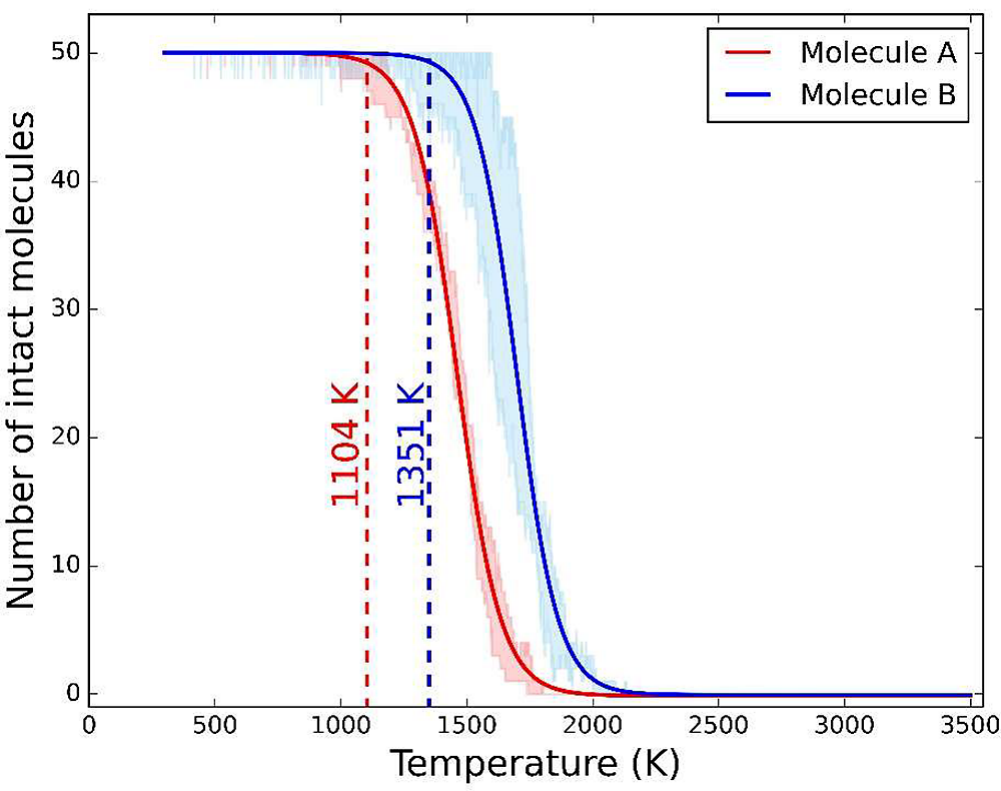
Evolution of the number of molecules A and B during the temperature ramp simulations. The shaded regions represent the range of intact molecule counts observed in three independent simulations and the solid lines represent fitted curves thereof. The dashed lines indicate the temperature at which thermo-oxidation starts to occur, i.e., the onset of degradation. The onset of degradation for molecule A is 1104 ± 48 and 1351 ± 135 K for molecule B.

We initially conducted temperature ramp simulations to determine the onset temperature for reactions within each model system. As shown in Figure 2, the number of intact molecules starts decreasing rapidly with increasing temperature around 1000 K as thermo-oxidation reactions begin. The number of intact molecules vs temperature data was fitted to an exponential decay equation from all three simulations for each molecule (solid lines in Figure 2). The functional form of this equation does not have any physical meaning since the last stage of the process, i.e., the number of intact molecules gradually approaching zero, is only due to the finite number of molecules in the model system. However, fitting the data enabled consistent determination of the temperature at which rapid thermo-oxidation began. Specifically, we identified the onset of degradation as the temperature where at least one bond dissociated causing the molecule to lose its intact structure.

As illustrated in Figure 2, our data indicates degradation onset temperatures of 1104 ± 48 K for molecule A and 1351 ± 135 K for molecule B, where the error ranges are the standard deviations of the three data sets. In an earlier simulation-based study of methoxy-containing lignin molecules, the temperature at which the first irreversible dissociation reactions were observed was reported to range from 1900 to 2300 K^22^. The lignin-derived molecules in our study had lower degradation onset temperatures (albeit not defined in the same way), likely because of the much lower heating rate of 4 K/ps vs 233 K/ps used in the previous study, and because replacing the methoxy group with a less stable *tert*-butyl group that leads to easier proton donation in reactions.

The purpose of the temperature ramp simulations was to identify the reaction products associated with the lowest energy barrier reaction pathways, so we performed preliminary simulations to determine the lowest temperature at which a statistically significant number of reactions occurred. The preliminary simulations were run at a range of temperatures between the degradation onset temperature determined from the temperature ramp simulations and 300 K below that onset temperature. The results showed that, at ∼100 K below the onset temperature, approximately 80% of the model molecules underwent degradation during the 1 ns simulation. Therefore, the thermo-oxidation simulations were run at a constant temperature of 1000 K for molecule A and 1250 K for molecule B. The results were then analyzed to identify reaction products and pathways, as described next.

**Figure 3:**
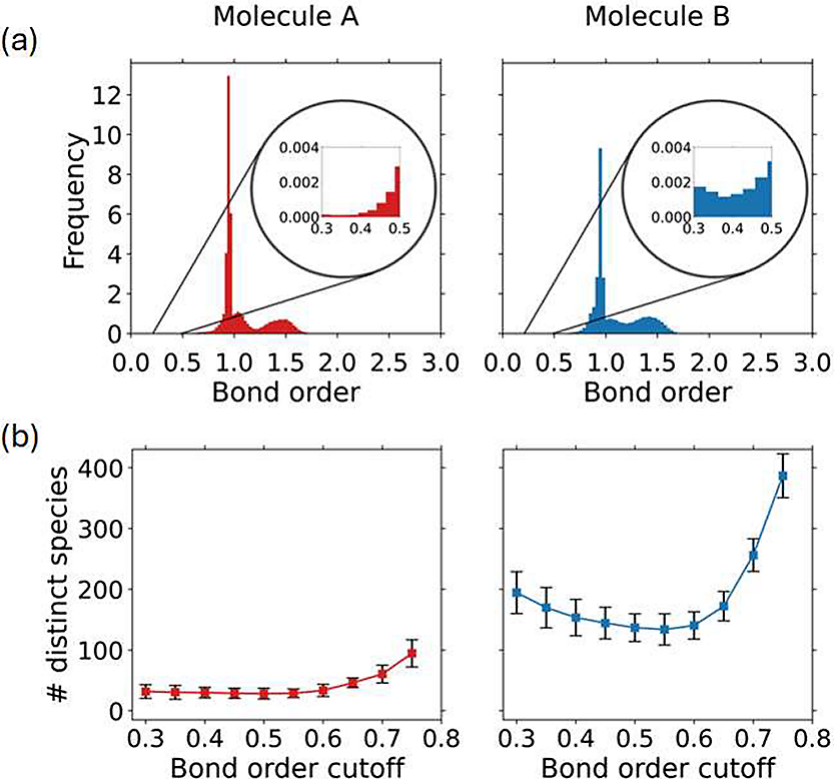
(a) Distributions of bond orders of all bonds during the thermo-oxidation simulations of molecules A and B, where the insets are close ups of the distributions between bond orders of 0.3 and 0.5 to show the relative number of bonds that were affected by the choice of the cutoff at 0.5. (b) Average number of distinct species identified at different bond order cutoffs for molecules A and B where the error bars represent the standard deviation calculated over three independent simulations.

Throughout the thermo-oxidation simulations, the chemical compositions of all species at each time step were determined based on the nonlinear bond-order relation proposed by van Duin et al.^26^ using the breadth-first search (BFS) algorithm.^36^ BFS is a graph traversal method used to find connected nodes where, in this case, each node is an atom and the connections are bonds. However, identifying chemical species requires first determining the criterion for a chemical bond between any two atoms. Since the bond order in ReaxFF is a continuous function, it was necessary to select a reasonably lower cutoff value for the bond order, above which a bond was considered present.^37,38^ The distributions of bond orders in Figure 3a show that most bond orders were between 0.3 and 1.8 for both molecules A and B. To determine a suitable cutoff, we analyzed how the number and chemistry of distinct species in the system varied as a function of the bond order cutoff, as illustrated in Figure 3b. For a too-small bond order cutoff, the number of distinct species was high and there were many unphysical bonding configurations. For a too-large bond order cutoff, there were again many distinct species, but they included fragmented molecules caused by bonds not identified where they should be. Since both high and low cutoffs resulted in many distinct species, resulting from either unphysical bonds (low cutoff) or fragmented molecules (high cutoff), we chose a bond order cutoff of 0.5 which yielded a low number of distinct, chemically reasonable species for both molecule A and B. A bond order cutoff of 0.5 has also been used previously in simulations of the oxidation pathway of hydrocarbons.^37^

Next, the reaction products were identified as the most prevalent species at the end of the thermo-oxidation simulations, where prevalence was determined based on the average number of each chemical species during the last 100 ps of the simulations. Then, once the products were known, we could trace them back to the reactants to outline their pathways. To identify the intermediate steps for each product, we tracked all the species that shared at least one atom with the product. The lifetime of each intermediate species, defined as the time duration for which the species exists, was calculated to eliminate those falling below a specified threshold of 12.5 ps (corresponding to 50 timesteps) during the entire simulation. This lifetime threshold was carefully chosen so that it only eliminated species that appeared and disappeared repeatedly due to relatively weak bonding. This process resulted in a list of intermediate species for each reaction product.

Finally, to understand the formation and dissociation of bonds that lead to the product from reactants through various intermediate species, the bond order of each bond in the original molecule was monitored over time. This approach enabled us to determine which bonds broke (bond order change from approximately 1 to 0) or transformed from single to double (bond order change from approximately 1 to 2) or vice versa (bond order change from approximately 2 to 1). The formation of new bonds throughout the entire simulation, contributing to the production of the final products, was also counted. This strategy was applied to molecules A and B to identify thermo-oxidation pathways, as described next.

**Figure 4:**
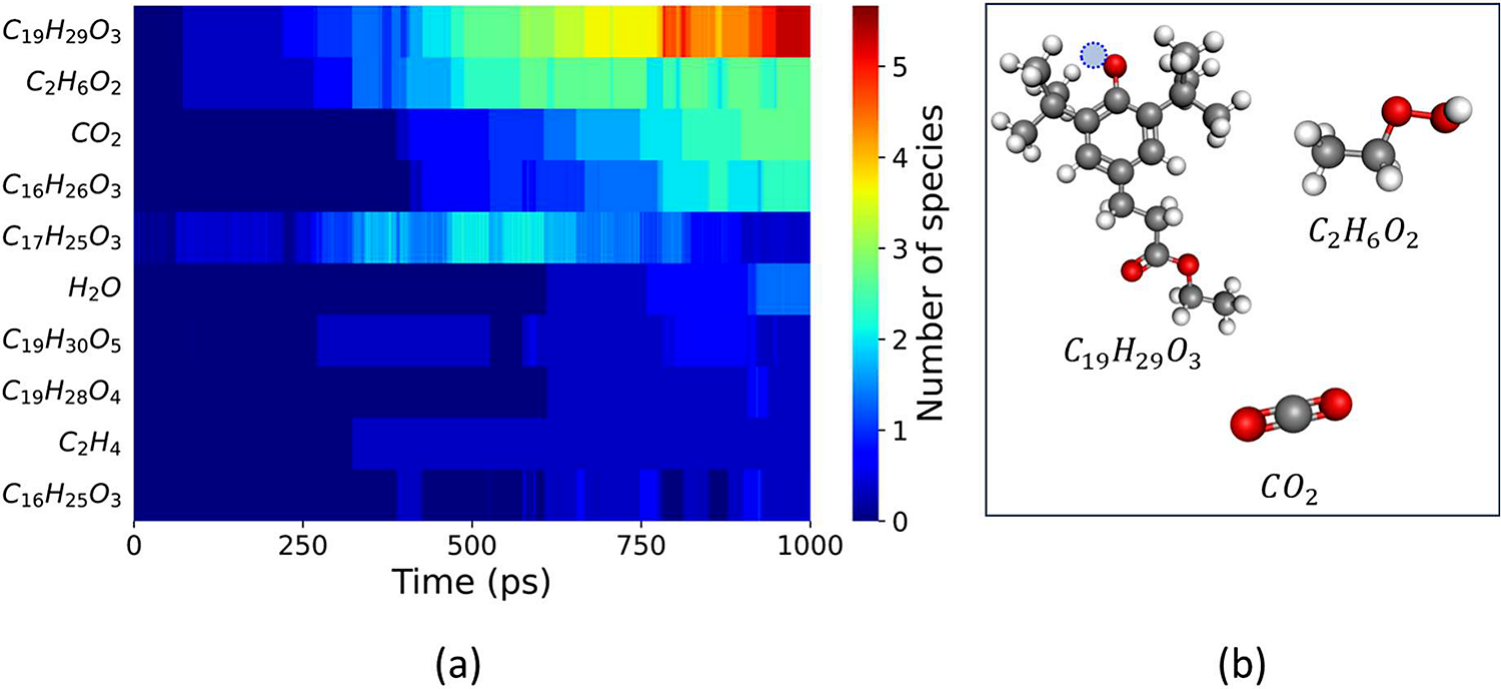
(a) Evolution of the ten most abundant species during the thermo-oxidation simulation of molecule A throughout the duration (1000 ps) of three independent simulations at 1000 K. (b) Structures of the three most prevalent reaction products from the thermo-oxidation of molecule A (C_29_H_30_O_3_) where a missing H atom is represented as a blue dashed circle.

The results for the ten most prevalent products during thermo-oxidation of molecule A are reported as a heatmap in Figure 4a, where the color reflects the number of each chemical species averaged over three independent simulations. This plot reveals C_19_H_29_O_3_, CO_2_, and C_2_H_6_O_2_ (ethyl hydroperoxide) as the dominant reaction products during the thermo-oxidation of molecule A (C_19_H_30_O_3_); the corresponding structures are shown in Figure 4b. C_19_H_29_O_3_ was observed when a single H atom detached from the hydroxyl group bonded to the aromatic ring. Degradation by thermo-oxidation, which is actually a combustion process, leads to products of different molecular sizes, typically to smaller species that eventually result in CO_2_ and H_2_O (water). Therefore, we chose to focus on the small products also for simplicity and tracked the reaction pathways leading to the formation of carbon dioxide and ethyl hydroperoxide.

**Figure 5:**
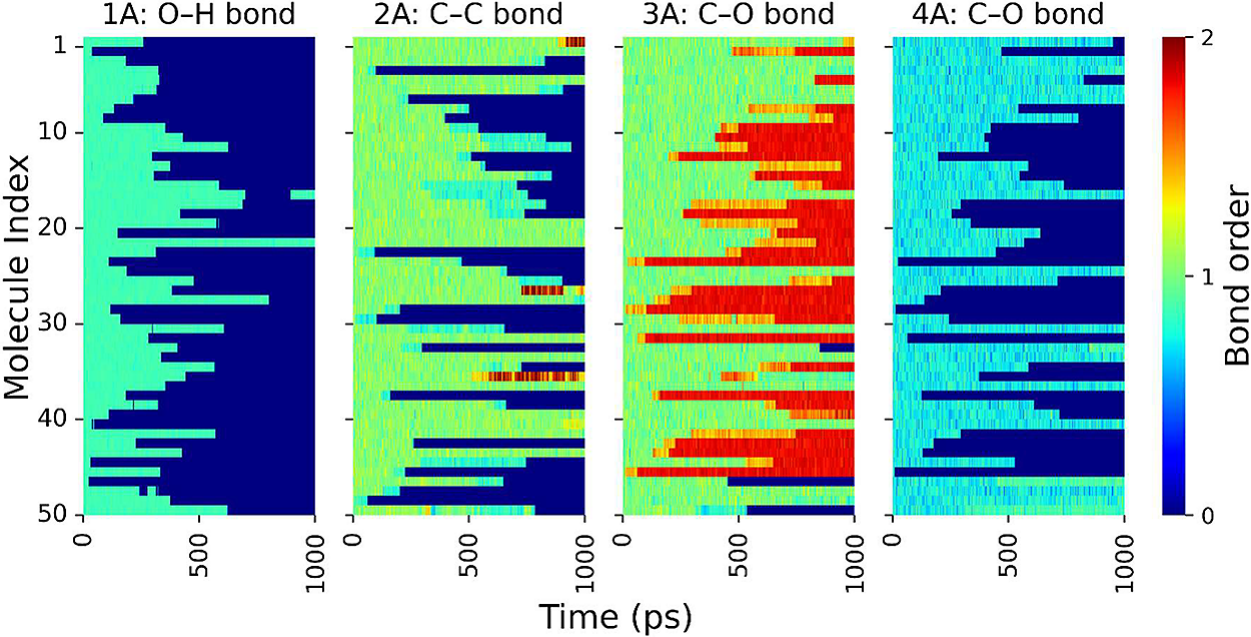
Evolution of bond orders for bonds 1A, 2A, 3A, and 4A (defined in Figure 1a) during the thermo-oxidation of molecule A. Each line represents one molecule in the model. Bond orders range from zero to two, approximately corresponding to no bonding, a single bond, or a double bond.

The four bonds that dissociated or formed most often during the production of prevalent reaction products of thermo-oxidation of molecule A are labeled in Figure 1a. The time evolution of the bond orders for these four key bonds is displayed as a series of heatmaps in Figure 5, where the color represents the bond order of each bond. Bond orders range here from zero to two, approximately corresponding to no bonding, a single bond, and a double bond. The results show that bonds 1A, 2A, and 4A dissociate, while bond 3A undergoes a transition from single to double bond.

**Figure 6:**
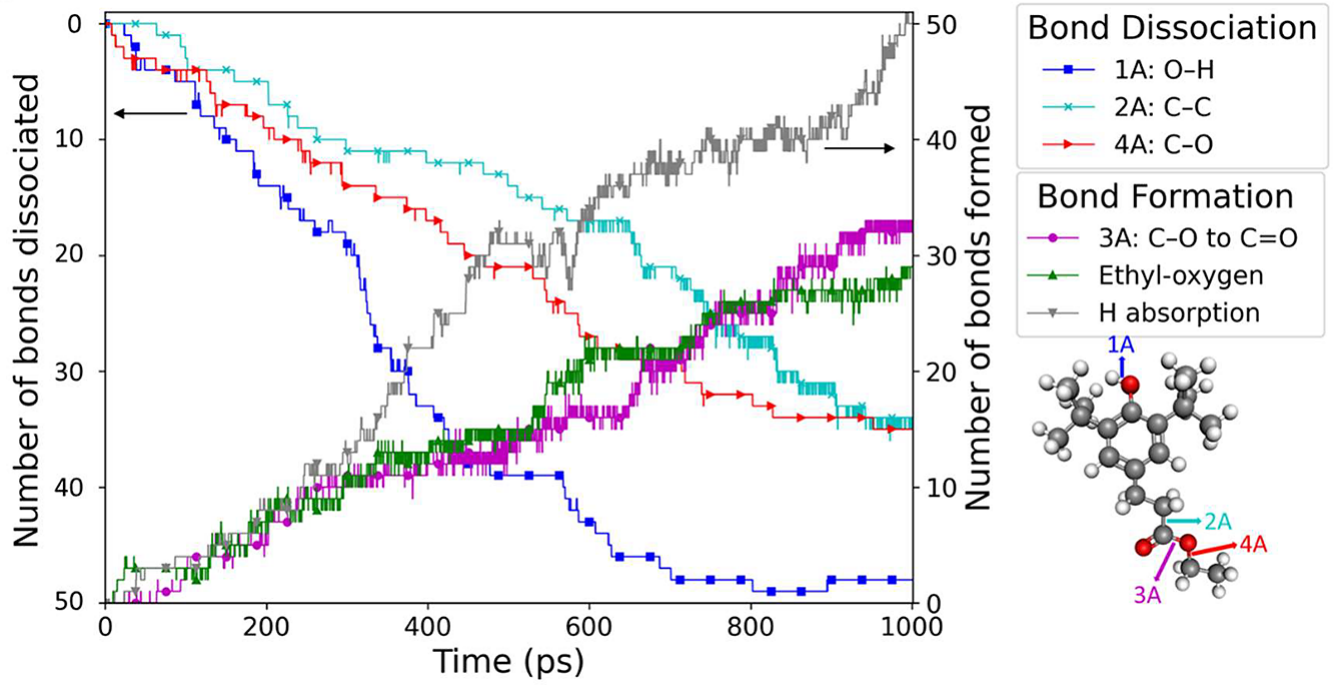
Evolution of the number of bonds 1A to 4A in molecule A. Bonds 1A, 2A, and 4A undergo dissociation, while bond 3A transitions from single to double. The formation of two new bonds is observed during the formation of ethyl hydroperoxide (C_2_H_6_O_2_): A C–O (ethyl-oxygen) bond is generated when the ethyl radical dissociates from molecule A and bonds with O_2_, and the absorption of an H atom leads to the creation of another bond between the H atom removed from 1A and the C_2_H_5_O_2_ radical.

We counted these four bonds, as well as two new bonds that formed during the reaction and were not part of the original molecule, at each time step. The result for molecule A is shown in Figure 6. Decreasing trends represent the dissociation of bonds, whereas increasing trends represent the formation of new bonds. Together, Figures 4 and 5 reveal the steps in the thermo-oxidation pathway of molecule A to form carbon dioxide and ethyl hydroperoxide. The dissociation of bond 1A leads to the removal of an H atom from the hydroxyl group. When bond 4A dissociates, the ethyl radical is detached. The transition of bond 3A from single to double occurs around the same time as the dissociation of bond 4A. Finally, bond 2A breaks, leading to the dissociation of carbon dioxide. Ethyl radicals, once separated from molecule A, bond with O_2_ and H atoms (originally from bond 1A), resulting in the formation of ethyl hydroperoxide.

**Figure 7:**
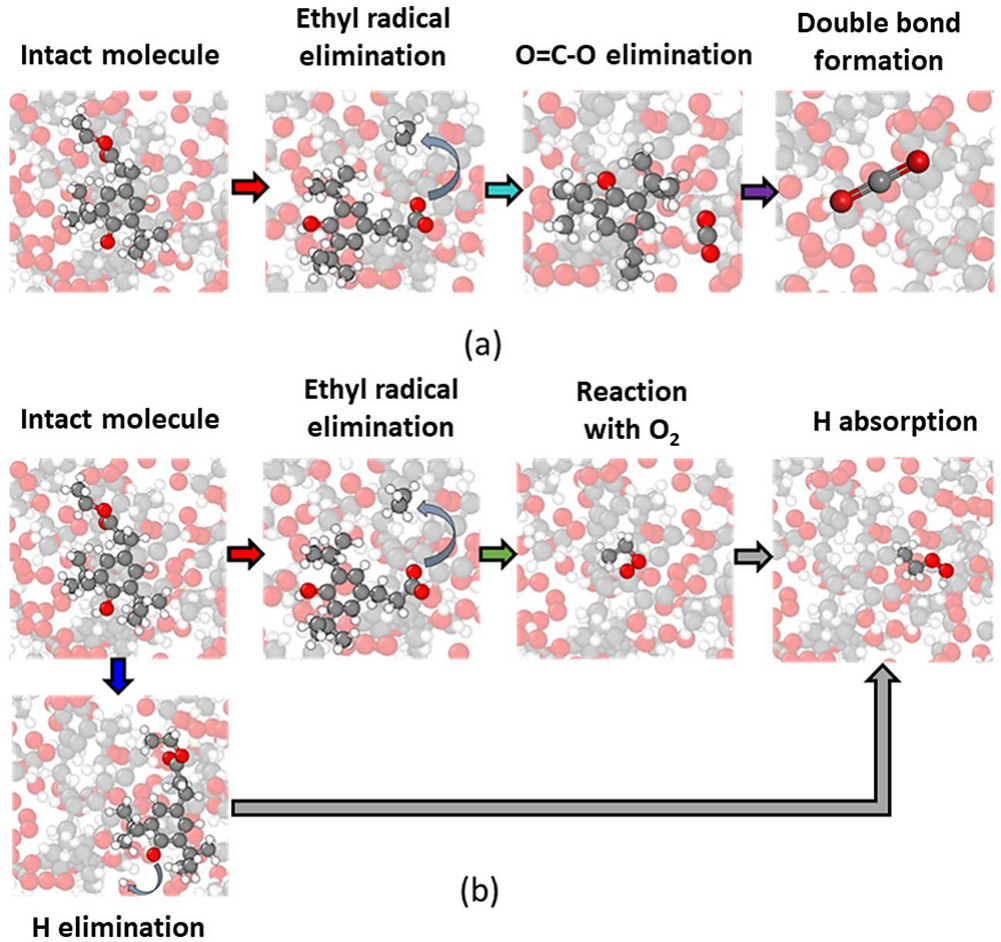
Snapshots showing the key steps of the formation of (a) CO_2_ and (b) C_2_H_6_O_2_ via thermo-oxidation of molecule A. The color of each arrow corresponds to the color of the respective curve in Figure 6. All atoms in the snapshots are faded except for those involved in the reactions of interest.

These reaction steps are shown graphically with representative molecules in Figure 7. Each snapshot corresponds to the breaking or forming of a specific bond, and the colors of the arrows after each step directly correspond to the curves of the same color shown in Figure 6. The first step in the pathways for the production of both CO_2_ and C_2_H_6_O_2_ was ethyl radical elimination by breaking the C–O bond between the ester oxygen and the ethyl group. This is corroborated by a previous DFT-based study of hydrothermal carbonization of lignite in which the first step in the reaction was C–O bond dissociation from the methyl group.^39^ In our simulations, the reaction pathway for CO_2_, shown in Figure 7a, started with an ethyl radical elimination, followed by the dissociation of a C–C bond to release an O=C–O radical, which finally becomes carbon dioxide. The pathway for ethyl hydroperoxide shown in Figure 7b started with the elimination of hydrogen and an ethyl radical. The radical bonds with O_2_ and the hydrogen atom to form ethyl hydroperoxide. As O_2_ was consumed, the numbers of both carbon dioxide and ethyl hydroperoxide increased.

**Figure 8:**
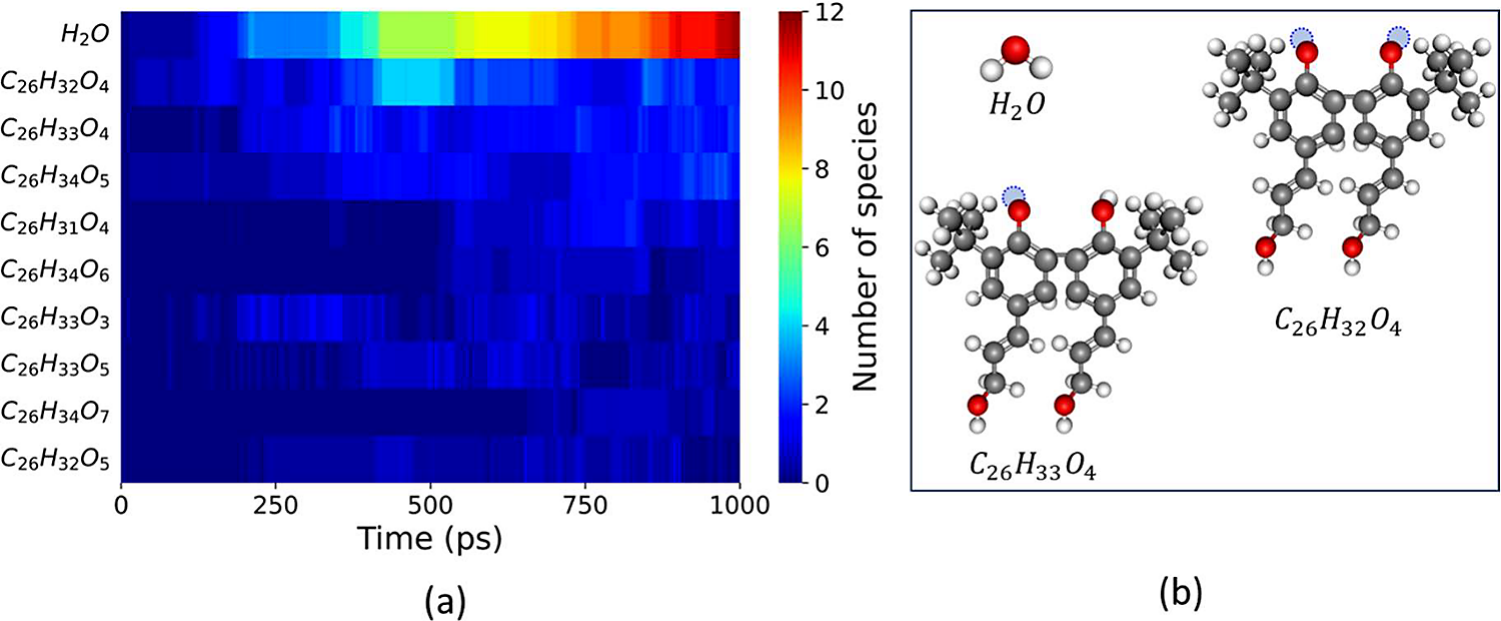
(a) Evolution of the ten most abundant species during the thermo-oxidation simulation of molecule B throughout the duration (1000 ps) of three independent simulations run 1250 K. (b) Structures of the three prevalent reaction products from the thermo-oxidation of molecule B (C_26_H_34_O_4_) where a missing H atom is represented as a blue dashed circle.

**Figure 9:**
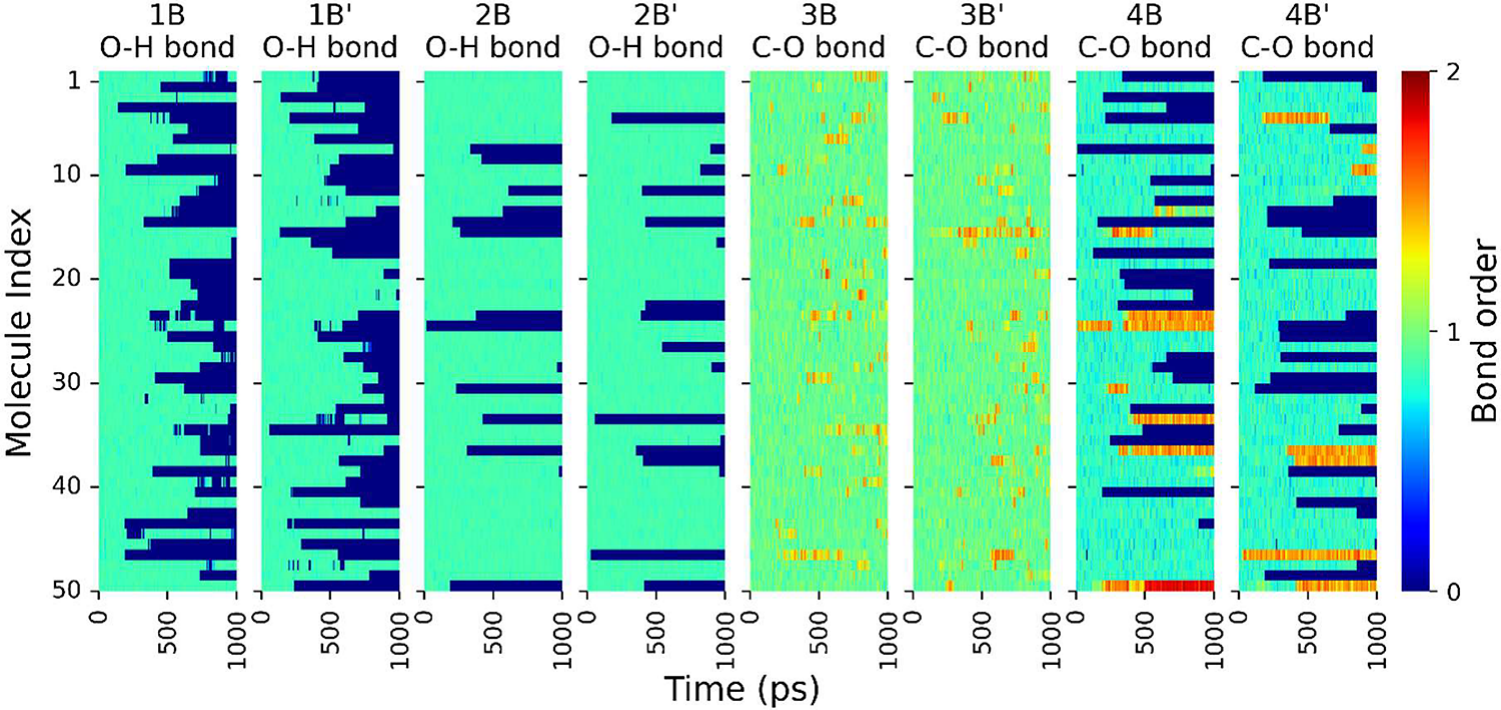
Evolution of bond orders for O–H and C–O bonds during the thermo-oxidation of molecule B. Bonds labeled 1B through 4B correspond to one aromatic ring, while 1B′ through 4B′ designate their equivalent bonds on the second ring (defined in Figure 1b). Each line represents one molecule in the model. Bond orders range from zero to two, approximately corresponding to no bonding, a single bond, or a double bond.

The same analysis approach was used for molecule B (C_26_H_34_O_4_). The time evolution of chemical species shown in Figure 8a reveals that the most prevalent products are H_2_O, C_26_H_32_O_4_, and C_26_H_33_O_4_; the corresponding structures are shown in Figure 8b. The latter two products were observed when H atoms detached from the aromatic ring (1B and 1B′). An analysis of the bond orders of selected bonds (labeled in Figure 1b) is shown in Figure 9. Here it can be observed that the O–H bond of the ring (phenolic OH) dissociates more readily than the O–H bond of the alkyl side chain (2B and 2B′). This is because, when an H atom dissociates from the phenolic O–H, the remaining structure is stabilized by aromatic resonance.^40^ The radical on oxygen is delocalized over the aromatic ring. However, this delocalization is not possible in the case of the alkyl side chain. With the C–O bond, the situation is reversed. Since the structure without H is stabilized by resonance, the ring C–O bonds are less prone to dissociation compared to the alkyl side chain C–O bonds.

The time evolution of the eight most frequently dissociated or formed bonds in molecule B is shown as a series of heatmaps in Figure 9. The dimer is symmetric such that there are four distinct bonds, each of which is present in one of the two monomers. These bonds are labeled as shown in Figure 1b, where a single prime symbol (′) is used to differentiate the bonds of the same type on the different monomers. Figure 9 shows that 1B/1B′ dissociates most readily. Closer observation from this plot reveals that, when the line for the 1B/1B′ bond in a given molecule transitions from green to blue (bond dissociation), the corresponding 3B/3B′ bond exhibits a reddish blip (from single to double bond). This observation indicates that, when a phenolic O–H bond dissociates, the radical oxygen is stabilized initially by forming a C=O double bond, which then delocalizes into the aromatic ring.

**Figure 10:**
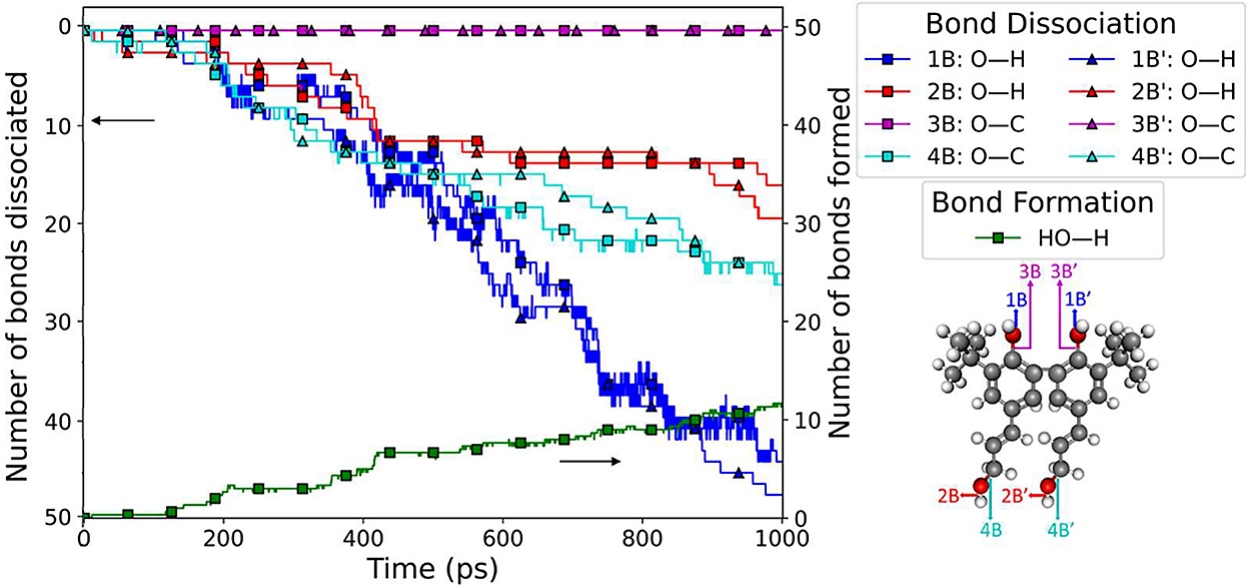
Evolution of the number of bonds1B to 4B and 1B′ to 4B′ in molecule B. Bonds 1B and 1B′ as well as 4B and 4B′ predominantly undergo dissociation while bonds 2B and 2B′ as well as 3B and 3B′ remain intact or experience limited dissociation. The formation of the HO–H bonds is observed during the formation of H_2_O.

The reaction analysis for molecule B was quantified as the number of different O–H and C–O bonds as well as the number of formed HO–H bonds in Figure 10. At the beginning of the simulation, there were 50 instances of each bond. As the thermo-oxidation began, the number of 1B/1B′, 2B/2B′, and 4B/4B′ bonds started to decrease. In contrast, the 3B/3B′ bonds did not dissociate, as mentioned earlier. Also, 2B/2B′ dissociated more slowly than 1B/1B′, which indicates that the hydrogen in the reaction predominantly came from the phenolic O–H group. This can be explained by previous DFT calculations that showed a phenolic O–H bond has a lower bond dissociation energy than a side chain O–H bond, while the phenolic C–OH bond has a higher bond dissociation energy than the side chain C–OH bond.^41^Figure 10 also shows that, initially, the HO–H bond was absent; however, as the simulation proceeded, the number of these bonds increased, indicating the formation of H_2_O.

**Figure 11:**
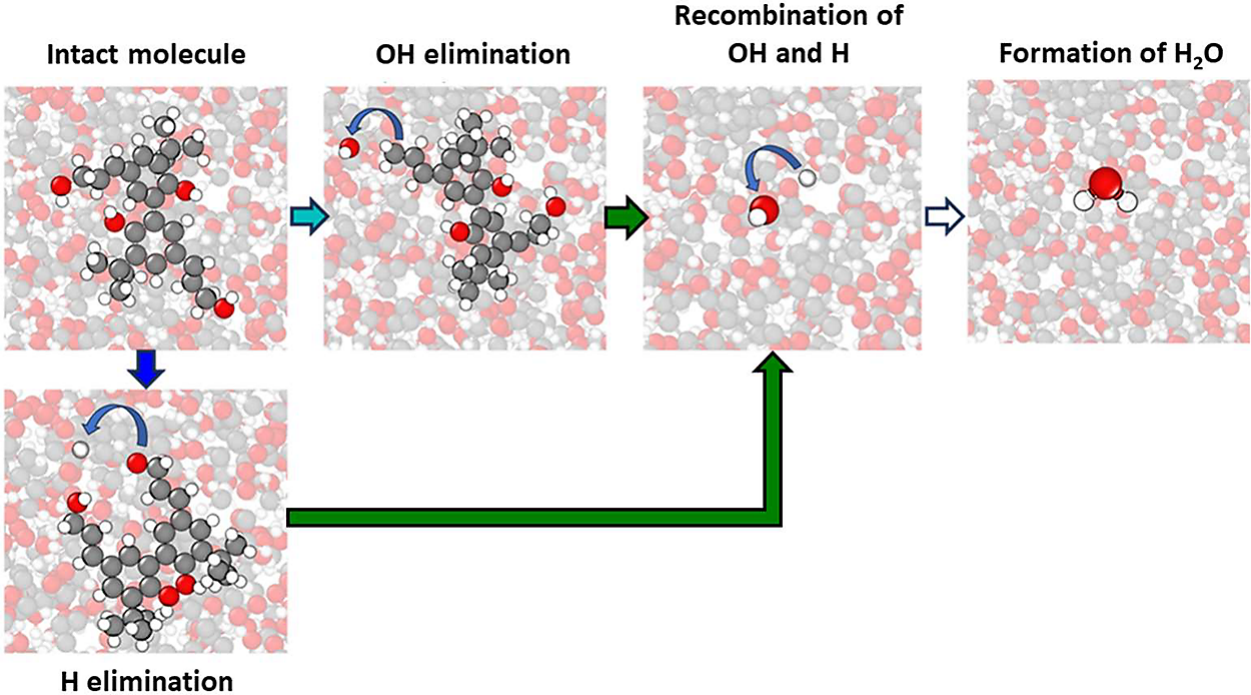
Snapshots showing the key steps of the formation of H_2_O from thermo-oxidation of molecule B. The color of each arrow corresponds to the color of the respective curve in Figure 10. All atoms in the snapshots are faded except for those involved in the reactions of interest.

Together, Figures 9 and 10 reveal the steps in the thermo-oxidation pathway of molecule B to form H_2_O. The dissociation of bonds 1B and 1B′ as well as 4B and 4B′ results in the release of an H atom and hydroxyl group, respectively, which then bond to form H_2_O. This process is illustrated in Figure 11 using representative molecules where each snapshot highlights the breaking or forming of a specific bond and the colors of the arrows in each step correspond to the curves of the same color in Figure 10. The reaction begins with the elimination of H atoms and OH radicals from molecule B, which then recombine to form H_2_O.

In the previous simulation-based research on similar model lignin molecules, the primary reaction products resulting from a thermo-oxidative environment were reported to be formaldehyde, water, and methanol.^22^ Formaldehyde and methanol were reported to form from the methoxy group in the 5–5 lignin model. In our study, we used structures obtained by hydrogenation, esterification, and butylation of a lignin monomer and a dimer. As the methoxy groups were replaced by butyl groups, the same reaction products were not detected in our study. In the lignin monomer model, alkoxy groups, such as methoxy, are commonly found and they contain a C–O bond, which is the weakest (247 kJ/mol).^42^ Replacing the methoxy group with a more stable *tert*-butyl group increases the reactivity of the O–H bond, leading to easier proton donation in reactions. Thus, the differences between the products identified in our simulations and the previous study are attributable to differences in molecular structures.

In another related study, ReaxFF MD was used to investigate the mechanisms of wheat straw pyrolysis^43^ where the authors observed an increase in small carbon products (C_0_–C_4_) at higher temperatures, while the number of medium-sized and large carbon products increased initially and then decreased over time. We observed a similar trend for oxidation of both molecules A and B. Figure 4a shows that the number of C_17_H_25_O_3_, a large carbon product, increased at first and then decreased, whereas the number of C_2_H_6_O_2_ and CO_2_ species continued to increase until the end of the simulation with molecule A. Similarly for molecule B shown in Figure 8a, the number of large carbon molecules, i.e., C_26_H_32_O_4_, C_26_H_33_O_4_, and C_26_H_34_O_5_, initially increased followed by a decrease in concentration, while the smaller product, H_2_O, increased monotonically over time. This agreement suggests that the products and pathways reported here may be more broadly applicable. This agreement suggests that the products and pathways reported here may be more broadly applicable.

Lastly, we compared the products and reaction pathways of molecules A and B. Comparing Figures 3a and 8a, while H_2_O is one of the reaction products for both molecules, it is much less abundant as a product of molecule A than of molecule B. On average, 3 and 12 H_2_O were formed during the thermo-oxidation of molecules A and B, respectively. We attribute the greater amount of H_2_O in the oxidation of molecule B to the fact that this molecule has four times as many hydroxyl groups as molecule A. These hydroxyl groups directly contributed to the formation of H_2_O. Most interesting for molecule B is that no oxygen was consumed during the simulation period of 1 ns to form the three main reaction products shown in Figure 8b, suggesting that the reactions were purely thermally driven. To test this suggestion, we conducted the same simulations (equilibration, three temperature ramp simulations, and three constant temperature simulations) of molecule B without oxygen and found that the reaction pathway was the same as that shown in Figure 11 from the simulations with oxygen. This result confirmed that water production from molecule B occurs through a purely thermal reaction pathway.

## Conclusions

In this work, we demonstrated a method to identify products and their pathways of thermo-oxidation reactions. We used reactive molecular dynamics to simulate the thermo-oxidation of two model structures of modified lignin referred to as molecule A (C_19_H_30_O_3_) and molecule B (C_26_H_34_O_4_). The chemical species generated during the thermo-oxidation were tracked to determine the dominant reaction products. The most relevant bonds were then tracked to identify the reaction pathways.

In the case of molecule A, we found that the prevalent three reaction products were C_19_H_29_O_3_, CO_2_, and C_2_H_6_O_2_. C_19_H_29_O_3_ was formed from H elimination from bond 1A. Dissociation of a C–C bond (2A) and a C–O bond (4A) led to the elimination of an O=C–O radical that finally formed carbon dioxide (CO_2_) by conversion of the C–O single bond (3A) into a double bond. The dissociation of the C–O bond (4A) resulted in the elimination of an ethyl radical from the reactant molecule, which then reacted with an O_2_ and an H to form ethyl hydroperoxide (C_2_H_6_O_2_).

For molecule B, H_2_O, C_26_H_32_O_4_, and C_26_H_33_O_4_ were the dominant reaction products. An O–H bond (1B and 1B′) located at the aromatic ring dissociated, releasing an H atom, while a C–O bond (4B and 4B′) located at the alkyl side chains (2B and 2B′) dissociated, releasing an OH radical. Then, the H atom and the OH radical recombined to form H_2_O.

For both molecules, we identified each step in the reaction by analyzing the bond dissociation and formation and observed the reaction using visualization software. In future work, we plan to conduct thermo-oxidation experiments with modified model lignin to directly compare and validate the computational findings. However, more generally, the developed methodology can be readily adapted to study the thermo-oxidation pathways of other organic molecules. The approach could then enable the engineering of synthesis processes to favor alternative desirable pathways and to assess the stability of molecules by the identification of the sites prone to dissociation, i.e., degradation. Ultimately, simulation-based tools such as those developed here can allow intentional modification of properties to create novel and stable materials.

## References

[ref1] Ouyang D.; et al. Light-driven lignocellulosic biomass conversion for production of energy and chemicals. iScience 2022, 25, 105221 10.1016/j.isci.2022.105221. 36262313 PMC9574509

[ref2] Pandey M. P.; Kim C. S. Lignin depolymerization and conversion: A review of thermochemical methods. Chem. Eng. Technol. 2011, 34, 29–41. 10.1002/ceat.201000270.

[ref3] Cho M.; Ko F. K.; Renneckar S. Impact of Thermal Oxidative Stabilization on the Performance of Lignin-Based Carbon Nanofiber Mats. ACS Omega 2019, 4, 5345–5355. 10.1021/acsomega.9b00278. 30949618 PMC6443214

[ref4] Letourneau D. R.; Volmer D. A. Mass spectrometry-based methods for the advanced characterization and structural analysis of lignin: A review. Mass Spectrom. Rev. 2023, 42, 144–188. 10.1002/mas.21716. 34293221

[ref5] Tardy B. L.; Lizundia E.; Guizani C.; Hakkarainen M.; Sipponen M. H. Prospects for the integration of lignin materials into the circular economy. Mater. Today 2023, 65, 122–132. 10.1016/j.mattod.2023.04.001.

[ref6] Kamm B.; Kamm M. Principles of biorefineries. Appl. Microbiol. Biotechnol. 2004, 64, 137–145. 10.1007/s00253-003-1537-7. 14749903

[ref7] Zhou N.; Thilakarathna W. P. D. W.; He Q. S.; Rupasinghe H. P. V. A Review: Depolymerization of Lignin to Generate High-Value Bio-Products: Opportunities, Challenges, and Prospects. Front. Energy Res. 2022, 9, 758744 10.3389/fenrg.2021.758744.

[ref8] da Cruz M. G. A.; et al. Electrochemical depolymerization of lignin in a biomass-based solvent. ChemSusChem 2022, 15, e202200718 10.1002/cssc.202201246. 35608798 PMC9545899

[ref9] da Cruz M. G. A.; et al. On the product selectivity in the electrochemical reductive cleavage of 2-phenoxyacetophenone, a lignin model compound. Green Chem. Lett. Rev. 2022, 15, 153–161. 10.1080/17518253.2022.2025462.

[ref10] Liu C.; Wu S.; Zhang H.; Xiao R. Catalytic oxidation of lignin to valuable biomass-based platform chemicals: A review. Fuel Process. Technol. 2019, 191, 181–201. 10.1016/j.fuproc.2019.04.007.

[ref11] Abdelaziz O. Y.; et al. On the Oxidative Valorization of Lignin to High-Value Chemicals: A Critical Review of Opportunities and Challenges. ChemSusChem 2022, 15, e202201232 10.1002/cssc.202201232. 36004569 PMC9825943

[ref12] Xiang Q.; Lee Y. Y. Oxidative cracking of precipitated hardwood lignin by hydrogen peroxide. Appl. Biochem. Biotechnol. 2000, 84–86, 153–162. 10.1385/ABAB:84-86:1-9:153. 10849786

[ref13] Hedges J. I.; Parker P. L. Land-derived organic matter in surface sediments from the Gulf of Mexico. Geochim. Cosmochim. Acta 1976, 40, 1019–1029. 10.1016/0016-7037(76)90044-2.

[ref14] Suzuki H.; et al. Wet oxidation of lignin model compounds and acetic acid production. J. Mater. Sci. 2006, 41, 1591–1597. 10.1007/s10853-006-4653-9.

[ref15] Yang L.; Seshan K.; Li Y. A review on thermal chemical reactions of lignin model compounds. Catal. Today 2017, 298, 276–297. 10.1016/j.cattod.2016.11.030.

[ref16] Petridis L.; Schulz R.; Smith J. C. Simulation analysis of the temperature dependence of lignin structure and dynamics. J. Am. Chem. Soc. 2011, 133, 20277–20287. 10.1021/ja206839u. 22035184

[ref17] Huda M. M.; Jahan N.; Rai N. Effect of water models on structure and dynamics of lignin in solution. AIP Adv. 2021, 11, 065024 10.1063/5.0047974.

[ref18] Mohan M.; et al. Prediction of solubility parameters of lignin and ionic liquids using multi-resolution simulation approaches. Green Chem. 2022, 24, 1165–1176. 10.1039/D1GC03798F.

[ref19] Aktulga H. M.; Pandit S. A.; van Duin A. C. T.; Grama A. Y. Reactive molecular dynamics: Numerical methods and algorithmic techniques. SIAM J. Sci. Comput. 2012, 34, C1–C23. 10.1137/100808599.

[ref20] Khajeh A.; et al. Thermal Decomposition of Tricresyl Phosphate on Ferrous Surfaces. J. Phys. Chem. C 2021, 125, 5076–5087. 10.1021/acs.jpcc.0c10789.

[ref21] Zhang Y. R.; van Duin A. C. T.; Luo K. H. Investigation of ethanol oxidation over aluminum nanoparticle using ReaxFF molecular dynamics simulation. Fuel 2018, 234, 94–100. 10.1016/j.fuel.2018.06.119.

[ref22] Beste A. ReaxFF Study of the Oxidation of Lignin Model Compounds for the Most Common Linkages in Softwood in View of Carbon Fiber Production. J. Phys. Chem. A 2014, 118, 803–814. 10.1021/jp410454q. 24428197

[ref23] Beste A. ReaxFF Study of the Oxidation of Softwood Lignin in View of Carbon Fiber Production. Energy Fuels 2014, 28, 7007–7013. 10.1021/ef501901p. 24428197

[ref24] BIOVIA. Dassault Systèmes, Material Studion, 20.1.0.27.28; Dassault Systèmes: San Diego, 2020.

[ref25] Plimpton S. Fast Parallel Algorithms for Short-Range Molecular Dynamics. J. Comput. Phys. 1995, 117, 1–19. 10.1006/jcph.1995.1039.

[ref26] van Duin A. C. T.; Dasgupta S.; Lorant F.; Goddard W. A. ReaxFF: A Reactive Force Field for Hydrocarbons. J. Phys. Chem. A 2001, 105, 9396–9409. 10.1021/jp004368u.

[ref27] Kim S.-Y.; et al. Development of a ReaxFF reactive force field for titanium dioxide/water systems. Langmuir 2013, 29, 7838–7846. 10.1021/la4006983. 23687907

[ref28] Senftle T. P.; et al. The ReaxFF reactive force-field: development, applications and future directions. npj Comput. Mater. 2016, 2, 15011 10.1038/npjcompumats.2015.11.

[ref29] Shin Y. K.; Kwak H.; Vasenkov A. V.; Sengupta D.; van Duin A. C. T. Development of a ReaxFF Reactive Force Field for Fe/Cr/O/S and Application to Oxidation of Butane over a Pyrite-Covered Cr2O3 Catalyst. ACS Catal. 2015, 5, 7226–7236. 10.1021/acscatal.5b01766.

[ref30] Ewen J. P.; et al. Substituent Effects on the Thermal Decomposition of Phosphate Esters on Ferrous Surfaces. J. Phys. Chem. C 2020, 124, 9852–9865. 10.1021/acs.jpcc.9b11787.

[ref31] Khajeh A.; et al. Statistical Analysis of Tri-Cresyl Phosphate Conversion on an Iron Oxide Surface Using Reactive Molecular Dynamics Simulations. J. Phys. Chem. C 2019, 123, 12886–12893. 10.1021/acs.jpcc.9b02394.

[ref32] Berendsen H. J. C.; van Gunsteren W. F.; DiNola A.; Haak J. R. Molecular dynamics with coupling to an external bath. J. Chem. Phys. 1984, 81, 3684–3690. 10.1063/1.448118.

[ref33] Nosé S. A unified formulation of the constant temperature molecular dynamics methods. J. Chem. Phys. 1984, 81, 511–519. 10.1063/1.447334.

[ref34] Hoover W. G. Canonical dynamics: Equilibrium phase-space distributions. Phys. Rev. A Gen. Phys. 1985, 31, 1695–1697. 10.1103/PhysRevA.31.1695. 9895674

[ref35] Stukowski A. Visualization and analysis of atomistic simulation data with OVITO–the Open Visualization Tool. Modell. Simul. Mater. Sci. Eng. 2010, 18, 015012 10.1088/0965-0393/18/1/015012.

[ref36] Cormen T. H.; Leiserson C. E.; Rivest R. L.; Stein C. Introduction to Algorithms; MIT Press, 2009; pp 594–602.

[ref37] Döntgen M.; et al. Automated discovery of reaction pathways, rate constants, and transition states using reactive molecular dynamics simulations. J. Chem. Theory Comput. 2015, 11, 2517–2524. 10.1021/acs.jctc.5b00201. 26575551

[ref38] Krep L.; et al. Efficient Reaction Space Exploration with ChemTraYzer-TAD. J. Chem. Inf. Model. 2022, 62, 890–902. 10.1021/acs.jcim.1c01197. 35142513

[ref39] Dang H.; et al. Study on chemical bond dissociation and the removal of oxygen-containing functional groups of low-rank coal during hydrothermal carbonization: DFT calculations. ACS Omega 2021, 6, 25772–25781. 10.1021/acsomega.1c03866. 34632233 PMC8495868

[ref40] Lowry T.; Richardson K. Mechanism and Theory in Organic Chemistry; Harper & Row, 1976.

[ref41] Dávalos J. Z.; Valderrama-Negrón A. C.; Barrios J. R.; Freitas V. L. S.; Ribeiro da Silva M. D. M. C. Energetic and Structural Properties of Two Phenolic Antioxidants: Tyrosol and Hydroxytyrosol. J. Phys. Chem. A 2018, 122, 4130–4137. 10.1021/acs.jpca.8b00457. 29616550

[ref42] Parkhurst H. J.; Huibers D.; Jones M. W. Production of phenol from lignin. Prepr. - Am. Chem. Soc. Div. Pet. Chem. 25: (3), , (1980).

[ref43] Liu Z.; Ku X.; Jin H. Pyrolysis Mechanism of Wheat Straw Based on ReaxFF Molecular Dynamics Simulations. ACS Omega 2022, 7, 21075–21085. 10.1021/acsomega.2c01899. 35755388 PMC9218979

